# microRNA-558 facilitates the expression of hypoxia-inducible factor 2 alpha through binding to 5′-untranslated region in neuroblastoma

**DOI:** 10.18632/oncotarget.9813

**Published:** 2016-06-03

**Authors:** Hongxia Qu, Liduan Zheng, Huajie Song, Wanju Jiao, Dan Li, Erhu Fang, Xiaojing Wang, Hong Mei, Jiarui Pu, Kai Huang, Qiangsong Tong

**Affiliations:** ^1^ Department of Pediatric Surgery, Union Hospital, Tongji Medical College, Huazhong University of Science and Technology, Wuhan 430022, Hubei Province, P. R. China; ^2^ Department of Pathology, Union Hospital, Tongji Medical College, Huazhong University of Science and Technology, Wuhan 430022, Hubei Province, P. R. China; ^3^ Clinical Center of Human Genomic Research, Union Hospital, Tongji Medical College, Huazhong University of Science and Technology, Wuhan 430022, Hubei Province, P. R. China

**Keywords:** neuroblastoma, microRNA-558, hypoxia-inducible factor 2 alpha, Argonaute 2, eukaryotic translation initiation factor 4E

## Abstract

Neuroblastoma (NB) is the most common extracranial solid tumor in childhood. Our previous studies have shown that hypoxia-inducible factor 2 alpha (HIF-2α), one member of the bHLH-PAS transcription factor family, facilitates the progression of NB under non-hypoxic conditions. However, the mechanisms underlying HIF-2α expression in NB still remain largely unknown. Herein, through analyzing the computational algorithm programs, we identified microRNA-558 (miR-558) as a crucial regulator of HIF-2α expression in NB. We demonstrated that miR-558 promoted the expression of HIF-2α at translational levels in NB cells through recruiting Argonaute 2 (AGO2). Mechanistically, miR-558 directly bound with its complementary site within 5′-untranslated region (5′-UTR) to facilitate the binding of AGO2 to eukaryotic translation initiation factor 4E (eIF4E) binding protein 1, resulting in increased eIF4E enrichment and HIF-2α translation. In addition, miR-558 promoted the growth, invasion, metastasis, and angiogenesis of NB cells *in vitro* and *in vivo*, and these biological features were rescued by knockdown of *AGO2*, *eIF4E*, or *HIF-2α*. In clinical NB specimens, miR-558, AGO2, and eIF4E were highly expressed and positively correlated with HIF-2α expression. Patients with high miR-558, HIF-2α, AGO2, or eIF4E levels had lower survival probability. Taken together, these results demonstrate that miR-558 facilitates the expression of HIF-2α through bindingto its 5′-UTR, thus promoting the tumorigenesis and aggressiveness of NB.

## INTRODUCTION

Neuroblastoma (NB), the most common malignant solid tumor derived from neural crest, accounts for 15% of all pediatric cancer mortality [1]. Although a few NB tumors can spontaneously regress or differentiate into benign ganglioneuroma, most of them present rapid progression or resistance to multimodal treatment, with the estimated 5-year event-free survival rate less than 50% [2], suggesting the urgency to explore the mechanisms underlying the progression of NB. Recent evidence shows that microRNAs (miRNAs), a class of small non-coding RNAs ranging from 22 to 25 nucleotides in length [3], exert crucial functions in tumorigenesis through inhibiting or activating gene expression at transcriptional and post-transcriptional levels. So far, a series of oncogenic miRNAs have been identified to participate in the initiation and progression of NB. For example, the miR-17-92 polycistronic cluster is highly expressed in NB tissues and serves as a marker for poor outcome of patients [4, 5]. miR-21, a well-known oncogenic miRNA, promotes the proliferation and decreases the chemosensitivity of NB cells [6]. In addition, miR-15a promotes the migration of NB cells through repressing the expression of reversion-inducing cysteine-rich protein with Kazal motifs [7]. Thus, it is necessary to further investigate the emerging functions of miRNAs to improve the therapeutics of NB.

Hypoxia-inducible factor 2 alpha (HIF-2α), one member of the bHLH-PAS transcription factor family, participates in regulation of gene expression through binding with promoters, and facilitates the growth, invasion, metastasis, and angiogenesis of cancers [8]. In many kinds of malignancies, such as renal cell carcinoma, breast carcinoma, and glioma, HIF-2α is highly expressed and associated with shorter survival or poor prognosis of patients [9]. It has been demonstrated that HIF-2α is highly expressed in well-vascularized and apparently non-hypoxic lesions of NB [10, 11]. In addition, a fraction of NB cells with intense HIF-2α immunostaining are the potential tumor initiating or stem cells under normoxic conditions, and are associated with unfavorable outcome [12]. Our previous studies have shown that miR-145 binds to the 3′-untranslated region (3′-UTR) of *HIF-2α* to repress its expression at post-transcriptional levels, and inhibits the progression of NB cells [10], demonstrating the important functions of HIF-2α in determining the aggressive behaviors of NB.

Although most studies of miRNAs focus on the binding sites within 3′-UTR in the last decade, recent studies have shown that endogenous miRNAs are able to recognize the 5′-untranslated region (5′-UTR) [13–16]. In the current study, we demonstrate, for the first time, that miR-558 directly binds to the 5′-UTR of *HIF-2α* to facilitate its translation through recruiting Argonaute 2 (AGO2) and increasing the binding of eukaryotic translation initiation factor 4E (eIF4E), thus promoting the growth, invasion, metastasis and angiogenesis of NB cells *in vitro* and *in vivo*. In addition, miR-558 was positively correlated with HIF-2α expression in clinical NB specimens, suggesting the oncogenic functions of miR-558 in the progression of NB through facilitating HIF-2α translation.

## RESULTS

### miR-558 facilitates the translation of HIF-2α in NB cells

**Figure 1:**
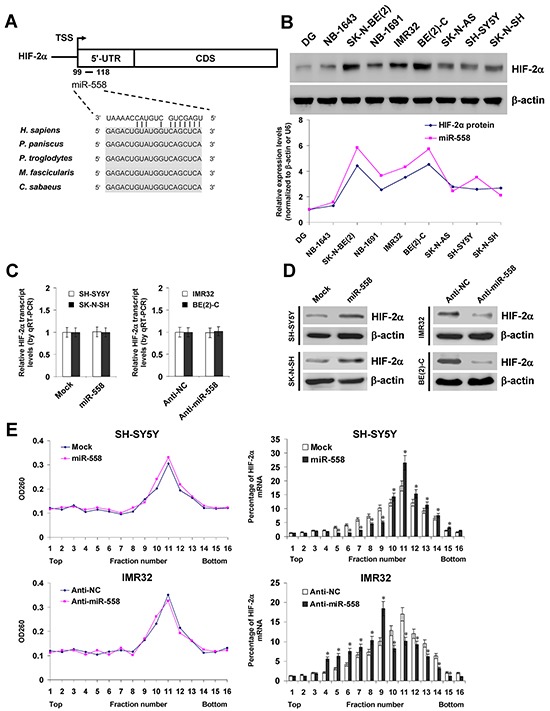
miR-558 facilitates the translation of HIF-2α in NB cells **A.** scheme of the potential binding site of miR-558 in the *HIF-2α* 5′-UTR, locating at bases 99-118 downstream the transcription start site (TSS). **B.** western blot and real-time qRT-PCR assays revealing the expression levels of HIF-2α and miR-558 in NB cell lines with [NB-1643, SK-N-BE(2), NB-1691, IMR32, BE(2)-C] or without *MYCN* amplification (SK-N-AS, SH-SY5Y, SK-N-SH) and normal dorsal ganglia (DG). **C.** and **D.** real-time qRT-PCR and western blot assays showing the transcript and protein levels of HIF-2α in NB cells transfected with empty vector (mock), miR-558 precursor, negative control inhibitor (anti-NC, 100 nmol/L), or anti-miR-558 inhibitor (100 nmol/L). **E.** sucrose gradient sedimentation assay indicating the distribution of *HIF-2α* transcripts to polysome fractions in NB cells transfected with mock, miR-558 precursor, anti-NC (100 nmol/L), or anti-miR-558 inhibitor (100 nmol/L). * *P*<0.01 vs. mock or anti-NC.

To investigate the hypothesis that miRNA may affect the translation of HIF-2α in NB, potential miRNA binding sites within its 5′-UTR were analyzed by computational algorithm programs miRWalk [17] and STarMir [18]. In the 5′-UTR of *HIF-2α*, there was one binding site of miR-558, locating at bases 99-118 downstream the transcription start site (TSS; Figure 1A). Higher miR-558 and HIF-2α levels were observed in NB cell lines, especially in *MYCN*-amplified SK-N-BE(2), IMR32, and BE(2)-C, than those in normal dorsal ganglia (Figure 1B). To determine the direct effects of miR-558 on HIF-2α expression in NB cells, we performed the miRNA over-expression and knockdown experiments. Stable transfection of miR-558 precursor into SH-SY5Y and SK-N-SH cells resulted in increase of miR-558 levels, than those stably transfected with empty vector (mock) (Supplementary Figure S1A). Meanwhile, transfection of anti-miR-558 inhibitor obviously down-regulated the expression of miR-558 in IMR32 and BE(2)-C cells, than those transfected with negative control (anti-NC) inhibitor (Supplementary Figure S1B). Real-time quantitative RT-PCR (qRT-PCR) and western blot assays demonstrated that ectopic expression or knockdown of miR-558 increased and decreased the protein levels of HIF-2α in cultured NB cells, respectively, without significant changes in *HIF-2α* transcript levels (Figure 1C and Figure 1D). In addition, sucrose gradient sedimentation assay indicated that over-expression or knockdown of miR-558 increased and decreased the distribution of *HIF-2α* transcripts to the heavy polysomes within fractions 10–12, respectively (Figure 1E). Since the analysis of miRNA databases from at least three independent sources revealed no potential binding site of miR-558 within the 3′-UTR of *HIF-2α*, and combining with the evidence that transfection of miR-558 did not affect the activity of *HIF-2α* 3′-UTR luciferase reporter (Supplementary Figure S1C and Supplementary Figure S1D), we ruled out the possibility that miR-558 precursor may directly facilitate the translation of HIF-2α via binding to the 3′-UTR. Overall, these results demonstrated that miR-558 considerably facilitated the translation of HIF-2α in NB cells.

### miR-558 binds to the complementary site within *HIF-2α* 5′-UTR in NB cells

**Figure 2:**
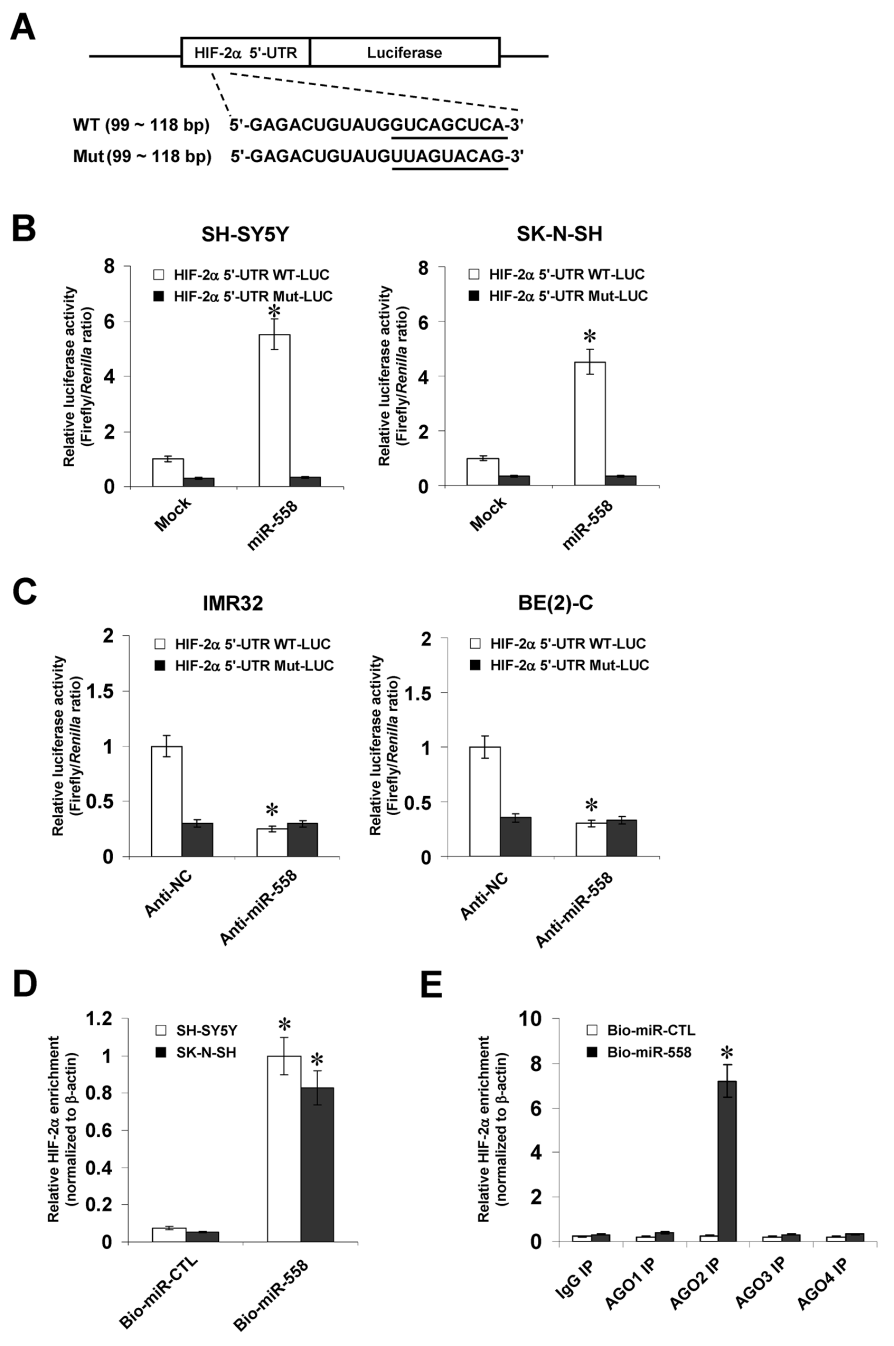
miR-558 binds to the complementary site within *HIF-2α* 5′-UTR in NB cells **A.** scheme and sequence of the intact miR-558 binding site (WT) and its mutation (Mut) within the *HIF-2α* 5′-UTR-luciferase reporter vectors. **B.** dual-luciferase assay indicating the activity of *HIF-2α* 5′-UTR reporter vector and its mutant in SH-SY5Y and SK-N-SH cells stably transfected with empty vector (mock) or miR-558 precursor. **C.** dual-luciferase assay showing the activity of *HIF-2α* 5′-UTR reporter vector and its mutant in IMR32 and BE(2)-C cells transfected with anti-NC (100 nmol/L) or anti-miR-558 (100 nmol/L) inhibitors. **D.** biotin-labeled pull-down and real-time qRT-PCR assays indicating the bound *HIF-2α* 5′-UTR in NB cells transfected with biotin-labeled control mimics (CTL) or miR-558 mimics. **E.** after immunoprecipitation with antibodies specific for AGO1, AGO2, AGO3, or AGO4, biotin-labeled pull-down and real-time qRT-PCR assays showing the bound *HIF-2α* 5′-UTR in NB cells transfected with biotin-labeled CTL or miR-558 mimics. * *P*<0.01 vs. mock, anti-NC, CTL, or IgG.

To determine whether or not miR-558 could facilitate HIF-2α translation by binding to its complementary site within 5′-UTR, the *HIF-2α* 5′-UTR-luciferase reporter or a mutant of miRNA seed recognition sequence (Figure 2A) were transfected into SH-SY5Y and SK-N-SH cells stably transfected with empty vector (mock) or miR-558 precursor. The firefly luciferase activity normalized to that of *Renilla* was significantly enhanced in NB cells stably transfected with miR-558 precursor (Figure 2B), and these effects were abolished by mutation of miR-558 binding site within the 5′-UTR of *HIF-2α* (Figure 2B). In addition, knockdown of miR-558 with anti-miR-558 inhibitor decreased the luciferase activity in IMR32 and BE(2)-C cells (Figure 2C), while mutation of miR-558 recognition site abolished these effects (Figure 2C). To further determine the direct binding of miR-558 to *HIF-2α* 5′-UTR, 3′-biotin-labeled miR-558 mimics were transfected into NB cells. RNA pull-down and real-time qRT-PCR assays indicated that transfection of miR-558 mimics into SH-SY5Y and SK-N-SH cells increased the levels of bound *HIF-2α* 5′-UTR, when compared to those transfected with biotin-labeled control miRNA mimics (Figure 2D). Since miRNAs bind to AGO proteins with very high affinity [19, 20], immunoprecipitation was performed using anti-AGO antibodies. The *HIF-2α* 5′-UTR was immunoprecipitated with antibody specific for AGO2, but not for AGO1, AGO3, or AGO4, in NB cells transfected with 3′-biotin-labeled miR-558 mimics (Figure 2E). These results indicated that miR-558 directly and specifically bound to the complementary site within *HIF-2α* 5′-UTR.

### miR-558 recruits AGO2 to facilitate the translation of HIF-2α in NB cells

**Figure 3:**
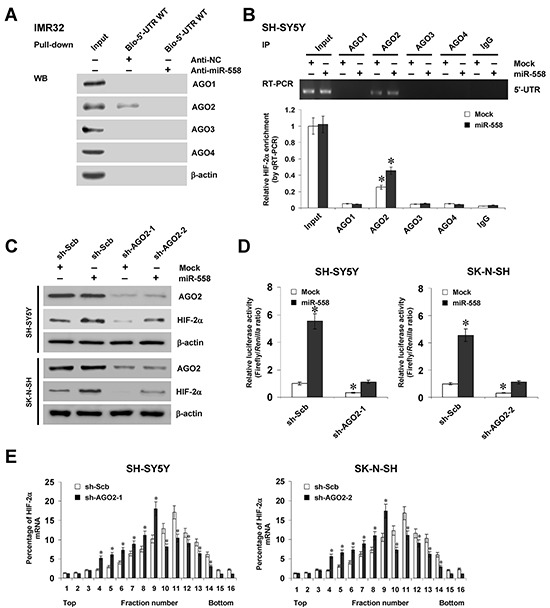
miR-558 recruits AGO2 to facilitate the translation of HIF-2α in NB cells **A.** RNA pull-down assay showing the binding of *HIF-2α* 5′-UTR to AGO1, AGO2, AGO3, or AGO4 in IMR32 cells transfected with anti-NC (100 nmol/L) or anti-miR-558 (100 nmol/L) inhibitors. **B.** RIP and real-time qRT-PCR assays indicating the binding of *HIF-2α* 5′-UTR to AGO1, AGO2, AGO3, or AGO4 in SH-SY5Y cells stably transfected with empty vector (mock) or miR-558 precursor. **C.** western blot assay showing the expression of AGO2 and HIF-2α in NB cells stably transfected with mock or miR-558 precursor, and those co-transfected with sh-Scb or sh-AGO2. **D.** dual-luciferase assay indicating the activity of *HIF-2α* 5′-UTR luciferase reporter in SH-SY5Y and SK-N-SH cells stably transfected with mock or miR-558 precursor, and those co-transfected with sh-Scb or sh-AGO2. **E.** sucrose gradient sedimentation assay showing the distribution of *HIF-2α* transcripts to the polysome fractions in NB cells transfected with sh-Scb or sh-AGO2. * *P*<0.01 vs. IgG, mock+sh-Scb, or sh-Scb.

To further investigate the functions of AGO protein in miR-558-facilitated translation of HIF-2α, the continuous 5′-UTR region of *HIF-2α* (+1/+510 bp relative to TSS) was assayed for AGO enrichment. Biotin-labeled RNA pull-down assay demonstrated the binding of *HIF-2α* 5′-UTR with AGO2, but not with AGO1, AGO3, or AGO4, which was significantly attenuated by knockdown of miR-558 in IMR32 cells (Figure 3A). RNA immunoprecipitation (RIP) and real-time qRT-PCR assays indicated that stable transfection of miR-558 precursor into SH-SY5Y cells resulted in increased binding of AGO2 to the *HIF-2α* 5′-UTR (Figure 3B). Importantly, western blot indicated that knockdown of *AGO2* via transfection of specific short hairpin RNA (shRNA) abolished the miR-558-faciliatated translation of HIF-2α in NB cells (Figure 3C). Dual-luciferase assay indicated that knockdown of *AGO2* prevented the SH-SY5Y and SK-N-SH cells from increased activity of *HIF-2α* 5′-UTR-luciferase reporter induced by stable transfection of miR-558 precursor (Figure 3D). In addition, sucrose gradient sedimentation assay revealed that knockdown of *AGO2* decreased the distribution of *HIF-2α* transcripts to the heavy polysomes within fractions 10–12 (Figure 3E). These results indicated that miR-558 recruited AGO2 to facilitate the translation of HIF-2α in NB cells.

### eIF4E contributes to miR-558-facilitated HIF-2α expression in NB cells

**Figure 4:**
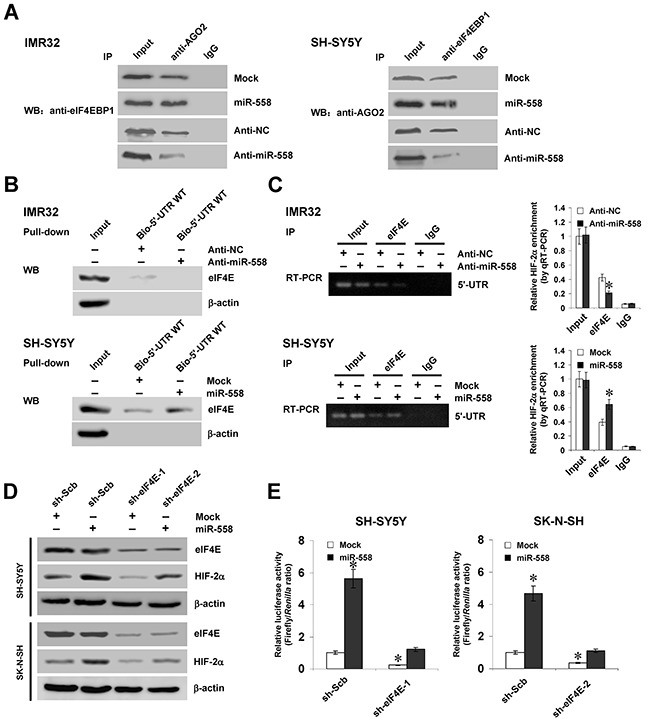
eIF4E contributes to miR-558-facilitated HIF-2α expression in NB cells **A.** Co-IP assay showing the binding of eIF4EBP1 to AGO2 in NB cells transfected with empty vector (mock), miR-558 precursor, anti-NC (100 nmol/L) or anti-miR-558 (100 nmol/L) inhibitors. **B.** and **C.** RNA pull-down and RIP assays indicating the binding of eIF4E to *HIF-2α* 5′-UTR in NB cells transfected with mock, miR-558 precursor, anti-NC (100 nmol/L) or anti-miR-558 (100 nmol/L) inhibitors. **D.** western blot assay showing the expression of eIF4E and HIF-2α in NB cells stably transfected with mock or miR-558 precursor, and those co-transfected with sh-Scb or sh-eIF4E. **E.** dual-luciferase assay indicating the activity of *HIF-2α* 5′-UTR luciferase reporter in SH-SY5Y and SK-N-SH cells stably transfected with mock or miR-558 precursor, and those co-transfected with sh-Scb or sh-eIF4E. * *P*<0.01 vs. anti-NC, mock, or mock+sh-Scb.

Since previous studies indicate the crucial functions of eIF4E in initial translation [21], and combining the evidence implicating the direct binding of AGO2 to eukaryotic translation initiation factor 4E binding protein 1 (eIF4EBP1) that represses the activity of eIF4E [22], we further explored the functions of eIF4E and eIF4EBP1 in miR-558-facilitated HIF-2α expression. Co-immunoprecipitation (Co-IP) and immunofluorescence assays revealed the endogenous binding of AGO2 to eIF4EBP1 in cultured NB cells (Figure 4A and Supplementary Figure S2). In addition, ectopic expression or knockdown of miR-558 increased and decreased the binding of AGO2 to eIF4EBP1, respectively (Figure 4A). Meanwhile, biotin-labeled RNA pull-down and RIP assays demonstrated the direct binding of eIF4E to *HIF-2α* 5′-UTR, which was significantly attenuated or enhanced by knockdown or over-expression of miR-558 in IMR32 and SH-SY5Y cells, respectively (Figure 4B and Figure 4C). Moreover, knockdown of *AGO2* decreased the enrichment of eIF4E on the 5′-UTR of *HIF-2α* in NB cells (Supplementary Figure S3A). Western blot indicated that knockdown of *eIF4E* via transfection of specific shRNAs abolished the miR-558-facilitated translation of HIF-2α in NB cells (Figure 4D). Dual-luciferase assay indicated that knockdown of *eIF4E* prevented the SH-SY5Y and SK-N-SH cells from increased activity of *HIF-2α* 5′-UTR-luciferase reporter induced by stable transfection of miR-558 precursor (Figure 4E). Sucrose gradient sedimentation assay revealed that knockdown of *eIF4E* decreased the distribution of *HIF-2α* transcripts to the heavy polysomes within fractions 10–12 (Supplementary Figure S3B and Supplementary Figure S3C). These results indicated that eIF4E contributed to miR-558-facilitated HIF-2α expression in NB cells.

### miR-558 promotes the growth, invasion, and angiogenesis of NB cells through facilitating HIF-2α expression *in vitro*

**Figure 5:**
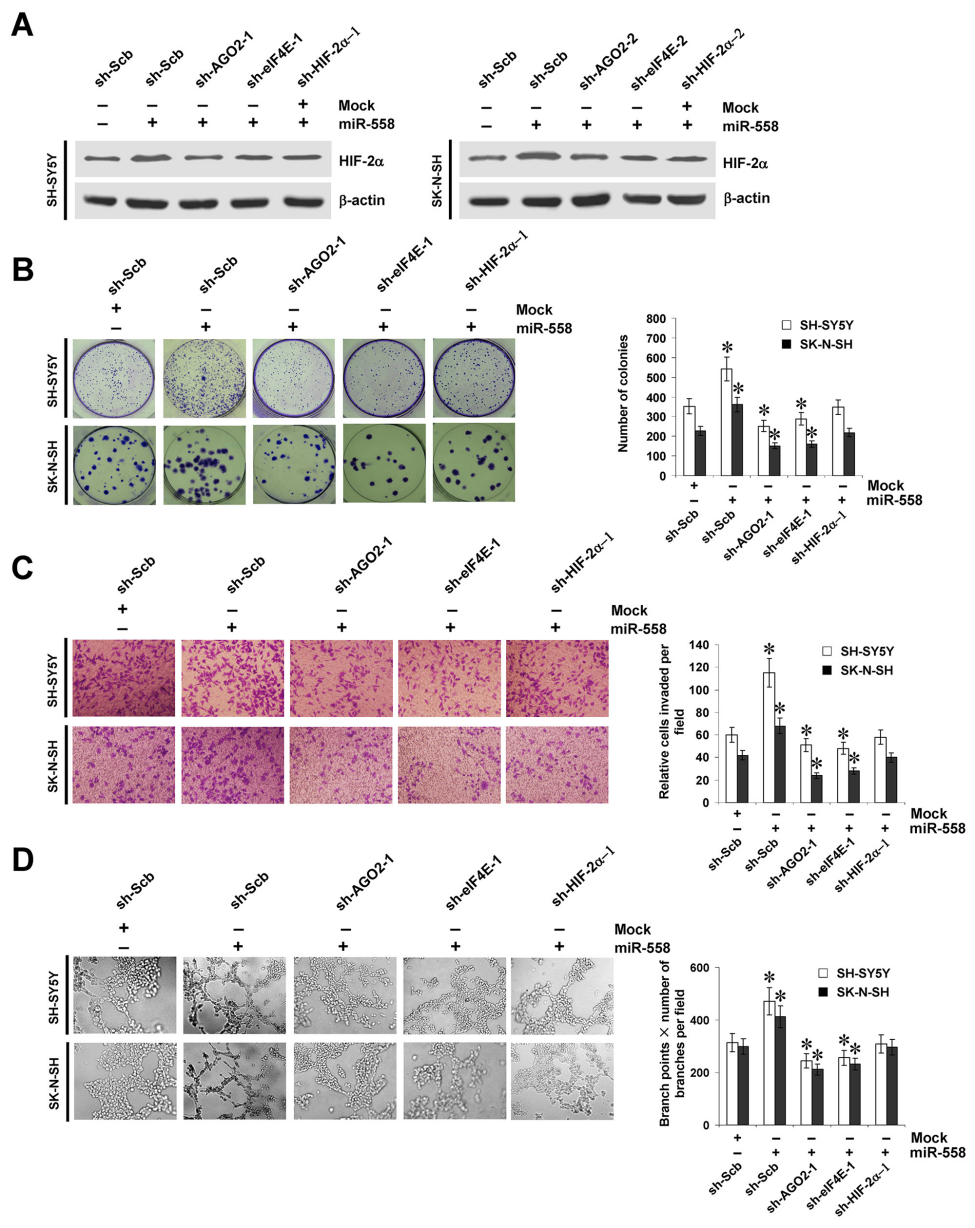
miR-558 promotes the growth, invasion, and angiogenesis of NB cells through facilitating HIF-2α expression *in vitro* **A.** western blot indicating the expression of HIF-2α in SH-SY5Y and SK-N-SH cells stably transfected with empty vector (mock) or miR-558 precursor, and those co-transfected with sh-Scb, sh-AGO2, sh-eIF4E, or sh-HIF-2α. **B.** representation (left) and quantification (right) of colony formation assay showing the growth of SH-SY5Y and SK-N-SH cells stably transfected with mock or miR-558 precursor, and those co-transfected with sh-Scb, sh-AGO2, sh-eIF4E, or sh-HIF-2α. **C.** representation (left) and quantification (right) of matrigel invasion assay indicating the invasion capability of SH-SY5Y and SK-N-SH cells stably transfected with mock or miR-558 precursor, and those co-transfected with sh-Scb, sh-AGO2, sh-eIF4E, or sh-HIF-2α. **D.** representation (left) and quantification (right) of tube formation assay showing the angiogenic capability of NB cells stably transfected with mock or miR-558 precursor, and those co-transfected with sh-Scb, sh-AGO2, sh-eIF4E, or sh-HIF-2α. * *P*<0.01 vs. mock+sh-Scb.

Since previous studies indicate that HIF-2α promotes the growth, invasion, and angiogenesis of tumor cells [10], we further investigated the effects of miR-558 over-expression and knockdown of *AGO2*, *eIF4E*, or *HIF-2α* on cultured NB cells. Western blot indicated that knockdown of *AGO2*, *eIF4E*, or *HIF-2α* rescued the miR-558-induced up-regulation of HIF-2α (Figure 5A). In colony formation assay, miR-558 over-expression promoted the growth of SH-SY5Y and SK-N-SH cells, when compared to those stably transfected with empty vector (mock) (Figure 5B). In matrigel invasion assay, NB cells stably transfected with miR-558 precursor presented an increased invasion capacity than mock cells (Figure 5C). The tube formation of endothelial cells was enhanced by treatment with the medium preconditioned by stable transfection of NB cells with miR-558 precursor (Figure 5D). In addition, transfection of shRNA specific for *AGO2*, *eIF4E*, or *HIF-2α* into SH-SY5Y and SK-N-SH cells rescued the increase in growth, invasion, and angiogenesis induced by stable over-expression of miR-558 (Figure 5B, Figure 5C, and Figure 5D). These results indicated that miR-558 remarkably increased the growth, invasion and angiogenesis of NB cells through facilitating HIF-2α expression *in vitro*.

### miR-558 facilitates the growth, metastasis, and angiogenesis of NB cells through increasing HIF-2α expression *in vivo*

**Figure 6:**
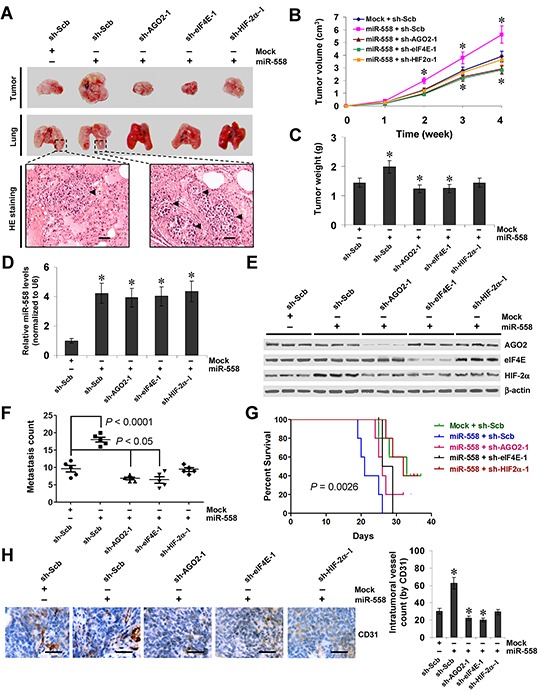
miR-558 promotes the growth, metastasis, and angiogenesis of NB cells through facilitating HIF-2α expression *in vivo* **A.** representative and HE staining images of xenograft tumors and lung metastatic colonies in athymic nude mice. **B.** tumor growth curve of SH-SY5Y (1×10^6^) stably transfected with empty vector (mock) or miR-558 precursor, and those co-transfected with sh-Scb, sh-AGO2, sh-eIF4E, or sh-HIF-2α in athymic nude mice (n=5 for each group), after hypodermic injection for 4 weeks. **C.** quantification of xenograft tumors formed by hypodermic injection of SH-SY5Y cells stably transfected with mock or miR-558 precursor, and those co-transfected with sh-Scb, sh-AGO2, sh-eIF4E, or sh-HIF-2α. **D.** and **E.** real-time qRT-PCR and western blot showing the expression of miR-558, AGO2, eIF4E, and HIF-2α in xenograft tumor tissues. **F.** and **G.** quantification of lung metastasis and Kaplan–Meier survival plots of nude mice with injection of SH-SY5Y cells (0.4×10^6^) stably transfected with mock or miR-558 precursor, and those co-transfected with sh-Scb, sh-AGO2, sh-eIF4E, or sh-HIF-2α via the tail vein (n=5 for each group). **H.** immunohistochemical staining (left) and quantification (right) of CD31 expression within tumors formed by hypodermic injection of SH-SY5Y cells stably transfected with mock or miR-558 precursor, and those co-transfected with sh-Scb, sh-AGO2, sh-eIF4E, or sh-HIF-2α. Scale bars: 100 μm. * *P*<0.001 vs. mock+sh-Scb.

We next investigated the efficacy of miR-558 over-expression and knockdown of *AGO2*, *eIF4E*, or *HIF-2α* against tumor growth, metastasis and angiogenesis *in vivo*. Stable transfection of miR-558 precursor into SH-SY5Y cells resulted in increased growth and tumor weight of subcutaneous xenograft tumors in athymic nude mice, when compared to those stably transfected with empty vector (mock) (Figure 6A, Figure 6B, and Figure 6C). Real-time qRT-PCR and western blot indicated the increased expression of miR-558 and HIF-2α in these xenograft tumor tissues (Figure 6D and Figure 6E). In the experimental metastasis studies, SH-SY5Y cells stably transfected with miR-558 precursor established statistically more lung metastatic colonies and lower survival probability than mock group (Figure 6A, Figure 6F, and Figure 6G). In addition, stable transfection of miR-558 precursor resulted in increase in CD31-positive microvessels and mean vessel density within tumors (Figure 6H). Stable transfection of shRNA specific for *AGO2*, *eIF4E*, or *HIF-2α* abolished these changes induced by stable over-expression of miR-558 (Figure 6A, Figure 6B, Figure 6C, Figure 6D, Figure 6E, Figure 6F, Figure 6G, and Figure 6H). These results suggested that miR-558 could facilitate the growth, metastasis and angiogenesis of NB cells through increasing HIF-2α expression *in vivo*.

### High expression of HIF-2α is positively correlated with miR-558, AGO2, or eIF4E levels in NB tissues

**Figure 7:**
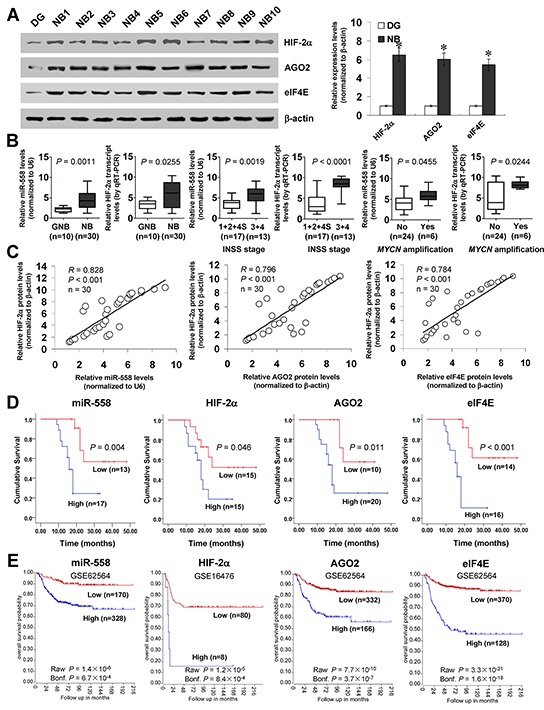
High expression of HIF-2α is positively correlated with miR-558, AGO2, or eIF4E levels in NB tissues **A.** western blot indicating the expression of HIF-2α, AGO2, and eIF4E in NB tissues (n=30) and normal dorsal ganglia (DG). **B.** real-time qRT-PCR assay showing the expression of miR-558 and HIF-2α in ganglioneuroblastoma (GNB, n=10) and NB (n=30), and their levels in NB cases with different INSS stage or *MYCN* amplification status. **C.** the correlation between HIF-2α protein and miR-558 transcript, AGO2 protein, or eIF4E protein levels in NB tissues (n=30). **D.** and **E.** Kaplan–Meier survival plots of 30 well-defined NB cases (stratified by median value) and public NB cohorts (stratified by the scan method and adjusted by Bonferroni correction) derived from GEO database and R2 microarray analysis and visualization platform (http://r2.amc.nl) indicating the survival probability of patients with high or low expression of miR-558, HIF-2α, AGO2, or eIF4E. * *P*<0.01 vs. DG.

Mining the publicly available datasets derived from Gene Expression Omnibus (GEO) database and R2: microarray analysis and visualization platform revealed higher expression of HIF-2α, AGO2, and eIF4E in NB tissues than those in normal dorsal ganglia (Supplementary Figure S4A). Higher expression of HIF-2α, AGO2, or eIF4E was observed in NB specimens with advanced international neuroblastoma staging system (INSS) stages (Supplementary Figure S4B) or *MYCN* amplification (Supplementary Figure S4C). In addition, western blot and real-time qRT-PCR were applied to measure the expression levels of HIF-2α, AGO2, eIF4E, and miR-558 in 30 NB specimens and normal dorsal ganglia. As shown in Figure 7A, higher expression of HIF-2α, AGO2, or eIF4E was observed in NB tissues than that in normal dorsal ganglia. Moreover, the expression of miR-558 or HIF-2α was higher in neuroblastic tumors with more aggressiveness (*P*=0.0011 and *P*=0.0255; Figure 7B), and in NB tissues with advanced INSS stage (*P*=0.0019 and *P*<0.0001; Figure 7B) or *MYCN* amplification (*P*=0.0455 and *P*=0.0244; Figure 7B). There was a positive correlation between HIF-2α protein levels and miR-558 expression (*R*=0.828, *P*<0.001), AGO2 expression (*R*=0.796, *P*<0.001), or eIF4E levels (*R*=0.784, *P*<0.001) in NB tissues (Figure 7C). Patients with high expression of miR-558 (*P*=0.004), HIF-2α (*P*=0.046), AGO2 (*P*=0.011), or eIF4E (*P*<0.001) had lower survival probability than those with low expression, respectively (Figure 7D). Kaplan–Meier survival plots of independent cohorts of NB cases derived from GEO database and R2 microarray analysis and visualization platform revealed that patients with high expression of miR-558 (*P*=1.4×10^−6^, Bonferroni-corrected *P*=6.7×10^−4^), HIF-2α (*P*=1.2×10^−5^, Bonferroni-corrected *P*=8.4×10^−4^), AGO2 (*P*=7.7×10^−10^, Bonferroni-corrected *P*=3.7×10^−7^; *P*=3.0×10^−6^, Bonferroni- corrected *P*=2.2×10^−4^; *P*=1.1×10^−12^, Bonferroni-corrected *P*=5.0×10^−10^), or eIF4E (*P*=3.3×10^−21^, Bonferroni-corrected *P*=1.6×10^−18^; *P*=5.7×10^−4^, Bonferroni-corrected *P*=4.1×10^−2^; *P*=6.4×10^−25^, Bonferroni-corrected *P*=3.0×10^−22^) had lower survival probability (Figure 7E and Supplementary Figure S5). These results indicated the high HIF-2α expression in primary NB tissues, which was positively correlated with miR-558, AGO2, and eIF4E levels.

## DISCUSSION

In recent years, many miRNA binding sites have been identified in the 5′-UTR, with higher density and conservation than those in the 3′-UTR [23]. In fact, miRNAs can bind to the 5′-UTR of messenger RNAs (mRNAs) [13–16]. Introduction of miRNA binding sites of let-7 into the 5′-UTR of luciferase reporter results in repressed translational efficiency in cervical cancer cells [13]. Meanwhile, miR-10a binds to the 5′-UTR of ribosome protein mRNA to enhance its translation [14]. In addition, miR-122 stimulates the translation of hepatitis C virus by enhancing the association of ribosomes with viral RNA at an early initiation stage [15]. Gain- and loss-of-function studies demonstrate that miR-346 elevates the levels of nuclear receptor interacting protein 1 by facilitating association of its mRNA with polysome fraction [16]. These results indicate that 5′-UTR-binding miRNAs can trigger both negative and positive responses in translation.

Human *HIF-2α* gene expression is mainly affected at the translation and post-translation levels [24]. Under normoxia, the HIF-2α protein is hydroxylated on two conserved proline residues, promoting its association with the von Hippel–Lindau ubiquitin ligase and subsequent proteasomal degradation [8]. In response to hypoxia, the hydroxylation of HIF-2α is abrogated, resulting in increased HIF-2α protein stability and activity to bind on DNA sequences, and transactivation of gene expression [8]. However, the mechanisms controlling the translation of HIF-2α still warrant further investigation. The translation process can be generally divided into three phases: initiation, elongation, and termination [21]. One of the translation initiating events is the binding of ribosomes on the 5′-cap structure of mRNA and scanning the 5′-UTR for AUG initiation codon [21]. In the current study, we demonstrated that miR-558 facilitated the translation of HIF-2α in NB cells through recruiting AGO2 and increasing the association of polysome fractions with *HIF-2α* 5′-UTR, without significant impacts on *HIF-2α* transcript levels. Our previous studies have shown that miR-145 is down-regulated in NB tissues, and suppresses the transcript and protein levels of *HIF-2α* in NB cells via the binding site within 3′-UTR [10]. Due to the up-regulation of miR-558 and down-regulation of miR-145 in NB, we believe that miR-558 is able to overcome the suppressive effects of miR-145 on HIF-2α expression in NB cells.

miR-558 is a recently identified miRNA that is repressed by ectopic expression of *MYCN* in NB cells, and its levels are not associated with *MYCN* amplification in high-risk NB samples [25]. In this study, we found higher miR-558 expression in NB tissues and cell lines with *MYCN* amplification. We believe that this discrepancy is due to different experimental design and heterogeneity of NB specimens, suggesting that further studies with a larger series of clinical tissues are warranted to investigate the exact impacts of *MYCN* amplification on miR-558 expression. Previous studies indicate that miR-558 markedly increases the proliferation, invasion, metastasis and angiogenesis of NB cells [25, 26], which was in line with the oncogenic functions of its host gene baculoviral IAP repeat containing 6 [27]. For miRNA-binding sites in the 5′-UTR, it is conceivable that the miRNA-induced silencing complex (miRISC) functions via specific miRNA–mRNA pairing [14] or miRISC-triggered recruitment of auxiliary repressor/activator complexes [28]. In this study, biotin-labeled miRNA pull-down, RNA pull-down, and RIP assays suggested that miR-558 directly bound to the 5′-UTR and recruited AGO2 to enhance the translation of HIF-2α. We demonstrated that knockdown of *HIF-2α* or *AGO2* prevented the miR-558-facilitated tumorigenesis and aggressiveness of NB cells *in vitro* and *in vivo*, suggesting that the miR-558 may exert its oncogenic functions, at least in part, through recruiting AGO2 to facilitate the HIF-2α expression in NB.

EIF4E, one rate-limiting component of the eIF4F translation initiation complex, is essential for the initiation of cap-dependent translation [21]. Previous studies show that eIF4E increases the translation of growth promoting and oncogenic proteins, such as c-Myc, cyclin D1, and vascular endothelial growth factor [29], and is associated with cellular growth, angiogenesis, and survival [29]. Over-expression of eIF4E is one of the early events in breast tumorigenesis [30], and is sufficient to induce transformation of cells in mouse tumor models [31–33]. Knockdown of *eIF4E* decreases the invasiveness and experimental metastasis of Ras-transformed fibroblasts [34]. Elevated eIF4E expression has been documented in many tumor tissues [35–37], and is associated with aggressiveness and poor outcome of patients [36, 37]. However, whether eIF4E is involved in the regulation of HIF-2α expression and tumorigenesis of NB still remains uncertain. In this study, our data demonstrated that eIF4E was necessary for miR-558-induced HIF-2α translation, which was associated with enrichment of eIF4E on the 5′-UTR. It has been indicated that the availability of eIF4E is negatively affected by the eIF4E-binding proteins, a family of proteins that sequester eIF4E by occupying the same binding site as eIF4G [21]. Our evidence showed that miR-558 facilitated the binding of AGO2 to eIF4EBP1 in NB cells, which is consistent with recent studies [22]. We believe that as important components of miRISC, miR-558/AGO2 complexes may bring in release of eIF4E from eIF4EBP1 to facilitate the HIF-2α expression, which warrants our further investigation.

In summary, we have shown that 5′-UTR-binding miR-558 facilitates the translation of HIF-2α via recruiting AGO2 and increasing the binding of eIF4E in NB cell lines. In addition, miR-558 promotes the growth, invasion, metastasis and angiogenesis of NB cells through facilitating HIF-2α expression *in vitro* and *in vivo*. This study extends our knowledge about the regulation of HIF-2α at the translational level by miRNAs and eIF4E, and suggests that miR-558 and eIF4E may be of potential values as novel therapeutic targets for human NB.

## MATERIALS AND METHODS

### Cell culture

Human NB cell lines NB-1643, SK-N-BE(2) (CRL-2271), NB-1691, IMR32 (CCL-127), BE(2)-C (CRL-2268), SK-N-AS (CRL-2137), SH-SY5Y (CRL-2266), and SK-N-SH (HTB-11), and human endothelial cell line HUVEC (CRL-1730) were purchased from Type Culture Collection of Chinese Academy of Sciences (Shanghai, China) and American Type Culture Collection (Rockville, MD). Cell lines were authenticated by the provider, used within 6 months after resuscitation of frozen aliquots, and grown in RPMI1640 medium (Life Technologies, Inc., Gaithersburg, MD) supplemented with 10% fetal bovine serum (Life Technologies, Inc.), penicillin (100 U/ml) and streptomycin (100 μg/ml). Cells were maintained at 37°C in a humidified atmosphere of 5% CO_2_ and applied for transfection.

### 5′-UTR-binding miRNA prediction and quantification

The miRNA binding sites within *HIF-2α* 5′-UTR were predicted using the computational algorithm programs miRWalk [17] and STarMir [18]. The levels of mature miR-558 in primary tissues and cell lines were determined using Bulge-Loop™ miRNAs qPCR Primer Set (RiboBio Co. Ltd, Guangzhou, China). After cDNA was synthesized with a miRNA-specific stem-loop primer, the quantitative PCR was performed with specific primers (Supplementary Table S1). The miRNA levels were normalized as to those of U6 snRNA.

### Over-expression and knockdown of miR-558

According to the pre-miR-558 (5′-TGAGCTGCTG TACCAAAAT-3′) sequence documented in miRNA Registry database [38], oligonucleotides encoding miR-558 precursor (Supplementary Table S2) were subcloned into the *BamH* I and *Xho* I restrictive sites of pcDNA3.1(−) (Genechem Co., Ltd, Shanghai, China), and verified by DNA sequencing. The plasmids pcDNA3.1 and pcDNA3.1-miR-558 were transfected into tumor cells, and stable cell lines were screened by administration of neomycin (Invitrogen, Carlsbad, CA). The miRNA inhibitors (antagomiR oligos) of miR-558 or negative control (RiboBio Co. Ltd) were transfected into confluent cells with Lipofectamine 2000 (Life Technologies, Inc.).

### Gene over-expression and knockdown

Human *HIF-2α* expression vector was previously described [10]. The oligonucleotides encoding shRNAs specific for *AGO2* (sh-AGO2), *eIF4E* (sh-eIF4E), or *HIF-2α* (sh-HIF-2α), and their scramble sequences were subcloned into GV102 (Genechem Co., Ltd, Shanghai, China; Supplementary Table S2). To restore the miRNA-induced up-regulation of HIF-2α, stable cell lines were transfected with sh-AGO2, sh-eIF4E, or sh-HIF-2α with Genesilencer Transfection Reagent (Genlantis, San Diego, CA).

### Western blot

Tissue or cellular protein was extracted with 1× cell lysis buffer (Promega, Madison, WI). Western blot was performed as previously described [10, 26, 39–47], with antibodies specific for HIF-2α, AGO1, AGO2, AGO3, AGO4, eIF4E, eIF4EBP1, and β-actin (Santa Cruz Biotechnology, Santa Cruz, CA). ECL substrate kit (Amersham, Piscataway, NJ) was used for the chemiluminscent detection of signals with autoradiography film (Amersham).

### Real-time quantitative RT-PCR

Total RNA was isolated with RNeasy Mini Kit (Qiagen Inc., Valencia, CA). The reverse transcription reactions were conducted with Transcriptor First Strand cDNA Synthesis Kit (Roche, Indianapolis, IN). The PCR primers for *HIF-2α* and *β-actin* were indicated in Supplementary Table S1. Real-time PCR with SYBR Green PCR Master Mix (Applied Biosystems, Foster City, CA) was performed using ABI Prism 7700 Sequence Detector (Applied Biosystems). The fluorescent signals were collected during extension phase, Ct values of the sample were calculated, and the transcript levels were analyzed by 2^−ΔΔCt^ method.

### Luciferase reporter assay

Human *HIF-2α* 5′-UTR luciferase reporter construct was kindly provided by Dr. Mayka Sanchez (Molecular Medicine Partnership Unit, Germany) [48]. The 3′-UTR luciferase reporter vector of *HIF-2α* was previously described [10]. Mutation of miR-558 binding site was performed with GeneTailor™ Site-Directed Mutagenesis System (Invitrogen) and PCR primers (Supplementary Table S2). Tumor cells were plated at 1×10^5^ cells/well on 24-well plates, and co-transfected with luciferase reporter vectors (30 ng) and *Renilla* luciferase reporter vector pRL-SV40 (10 ng, Promega). Twenty-four hrs post-transfection, firefly and *Renilla* luciferase activity was consecutively measured, according to the dual-luciferase assay manual (Promega). For 5′-UTR and 3′-UTR luciferase reporter activity, the luciferase signal was normalized by firefly/*Renilla* and *Renilla*/firefly ratio, respectively.

### Sucrose gradient sedimentation

The sedimentation of polysomal fractions was performed as previously described [16, 49]. Briefly, tumor cells were treated with 100 μg/ml of cycloheximide (Sigma, St. Louis, MO) for 5–10 min. Cell extracts were layered on top of 15–30% (w/v) linear sucrose gradient. After centrifugation at 40,000 ×g for 2 hrs at 4°C, fractions were collected using a piston-gradient fractionator (Biocomp, Fredericton, Canada). The polysome profiles were monitored by absorbance of light with a wavelength of 260 nm (A260). The polysome-bound transcripts were extracted and detected by reverse transcription and real-time qRT-PCR.

### Biotin-miRNA pull-down assay

The 3′-biotin-labeled miRNA mimics were synthesized and transfected into 1×10^6^ tumor cells as preciously described [50]. Briefly, cell lysis was mixed with streptavidin beads for 1 hr. The RNA bound to the beads (pulled-down RNA) or from the extract (input RNA) was isolated using the RNeasy Mini Kit (Qiagen Inc.). The retrieved transcript levels were detected by reverse transcription and real-time qRT-PCR, and normalized to those of the housekeeping gene (*β-actin*).

### RNA pull-down assay

Human *HIF-2α* 5′-UTR was *in vitro* transcribed with TranscriptAid T7 High Yield Transcription Kit (Thermo Fisher Scientific, Inc., Grand Island, NY). Biotin-labeled RNA pull-down was performed as previously described [51]. Briefly, cellular cytoplasmic proteins were extracted using the NE-PER Nuclear and Cytoplasmic Extraction Reagents (Thermo Fisher Scientific, Inc.), and incubated with biotin-labeled RNAs and streptavidin agarose beads (Invitrogen). The retrieved protein was detected by western blot.

### RNA immunoprecipitation

The RIP assay was performed according to the manufacture's instructions of Magna RIP™ RNA-Binding Protein Immunoprecipitation kit (Millipore, Billerica, MA). The PCR primers specific for *HIF-2α* 5′-UTR were indicated in Supplementary Table S1. Real-time PCR with SYBR Green PCR Master Mix was performed using ABI Prism 7700 Sequence Detector. The amount of immunoprecipitated RNA was calculated in reference to a standard curve and normalized to input RNA.

### Co-immunoprecipitation

Co-immunoprecipitation was performed as previously described [52], with antibodies specific for AGO2 and eIF4EBP1 (Upstate Biotechnology, Temacula, CA). The bead-bound proteins were released by boiling the protein A-Sepharose beads (Santa Cruz Biotechnology) in 1×SDS-PAGE loading buffer and analyzed by western blot.

### Immunofluorescence assay

Tumor cells were plated on coverslips, permeabilized with 0.3% Triton X-100, and blocked with 5% milk for 1 hr. Cells were incubated at 4°C overnight with antibodies specific for AGO2 or eIF4EBP1 (Santa Cruz Biotechnology; 1:200 dilutions). Then, cells were incubated with Alexa Fluor 594 goat anti-rabbit IgG (1:1000 dilution), stained with 4′,6-diamidino-2-phenylindole (DAPI, 300 nmol/L) to visualize nuclei, and photographed under a microscope.

### Colony formation assay

Tumor cells were seeded at a density of 300 cells/ml on 35-mm dishes. Colony formation assay was performed as previously described [41, 53]. Positive colony formation (more than 50 cells/colony) was counted. The survival fraction of cells was expressed as the ratio of plating efficiency of treated cells to that of untreated control cells.

### Cell invasion assay

Matrigel invasion assay was performed using membranes coated with Matrigel matrix (BD Science, Sparks, MD). Homogeneous single cell suspensions (1 × 10^5^ cells/well) were added to the upper chambers and allowed to invade for 24 hrs at 37°C in a CO_2_ incubator. Invaded cells were stained with 0.1% crystal violet for 10 min at room temperature and examined by light microscopy. Quantification of invaded cells was performed according to published criteria [10, 26, 39, 42–47, 51, 54].

### Tube formation assay

Fifty microliters of growth factor-reduced matrigel were polymerized on 96-well plates. HUVECs were serum starved in RPMI1640 medium for 24 hrs, suspended in RPMI1640 medium preconditioned with tumor cells, added to the matrigel-coated wells at the density of 5×10^4^ cells/well, and incubated at 37°C for 18 hrs. Quantification of anti-angiogenic activity was calculated by measuring the length of tube walls formed between discrete endothelial cells in each well relative to the control [10, 26, 39, 43, 45–47, 51, 54].

### *In vivo* growth, metastasis and angiogenesis assay

All animal experiments were approved by the Animal Care Committee of Tongji Medical College (approval number: Y20080290). For the *in vivo* tumor growth studies, 2-month-old male BALB/c nude mice (n=5 per group) were injected subcutaneously in the upper back with 1×10^6^ tumor cells stably transfected with mock, miR-558 precursor, sh-AGO2, sh-eIF4E, or sh-HIF-2α vectors. One month later, mice were sacrificed and examined for tumor weight and angiogenesis. The experimental metastasis (0.4×10^6^ tumor cells per mouse, n=5 per group) studies were performed with blindly randomized 2-month-old male BALB/c nude mice as previously described [10, 26, 39, 42, 43, 45–47, 51].

### Immunohistochemistry

Immunohistochemical staining was performed as previously described [10, 26, 39, 42, 43, 45–47, 51], with antibody specific for CD31 (Santa Cruz Biotechnology; 1:200 dilution). The negative controls included parallel sections treated with omission of the primary antibody, in addition to an adjacent section of the same block in which the primary antibody was replaced by rabbit polyclonal IgG (Abcam Inc.) as an isotype control. The degree of positivity was initially classified according to the percentage of positive tumor cells.

### Patient tissue samples

Approval to conduct this study was obtained from the Institutional Review Board of Tongji Medical College. Fresh specimens from 30 well-established primary NB cases and 10 ganglioneuroblastoma (GNB) patients were obtained from the Department of Pediatric Surgery, Union Hospital, Tongji Medical College, and stored at −80°C until use [26, 46]. The pathological diagnosis was confirmed by at least two pathologists. Based on the Shimada classification system, including the mitosis karyorrhexis index, degree of neuroblastic differentiation and stromal maturation and patient's age, 14 NB patients were classified as having favorable histology and 16 as having unfavorable histology. According to the INSS, four NB patients were classified as stage 1, nine as stage 2, nine as stage 3, four as stage 4, and four as stage 4S. The protein and RNAs of normal human dorsal ganglia were obtained from Clontech (Mountain View, CA).

### Statistical analysis

Unless otherwise stated, all data were shown as mean ± standard error of the mean (SEM). The χ^2^ analysis and Fisher exact probability analysis were applied for comparison among the gene expression and individual clinicopathological feature. Pearson's coefficient correlation was applied for analyzing the relationship between HIF-2α and miR-558, AGO2, or eIF4E levels. The Kaplan-Meier method was used to estimate survival rates, and log-rank test and Bonferroni correction were used to assess survival difference. Difference of tumor cells was determined by *t* test or analysis of variance (ANOVA).

## SUPPLEMENTARY FIGURES AND TABLES


