# Supplementary material for: microRNA-558 facilitates the expression of hypoxia-inducible factor 2 alpha through binding to 5′-untranslated region in neuroblastoma
Source: Oncotarget. 2016 Jun 3;7(26):40657–73. doi: 10.18632/oncotarget.9813 (PMC5130034; doi:10.18632/oncotarget.9813)
Supplement: Supplementary file 1 [file oncotarget-07-40657-s001.pdf]

## SUPPLEMENTARY FIGURES AND TABLES

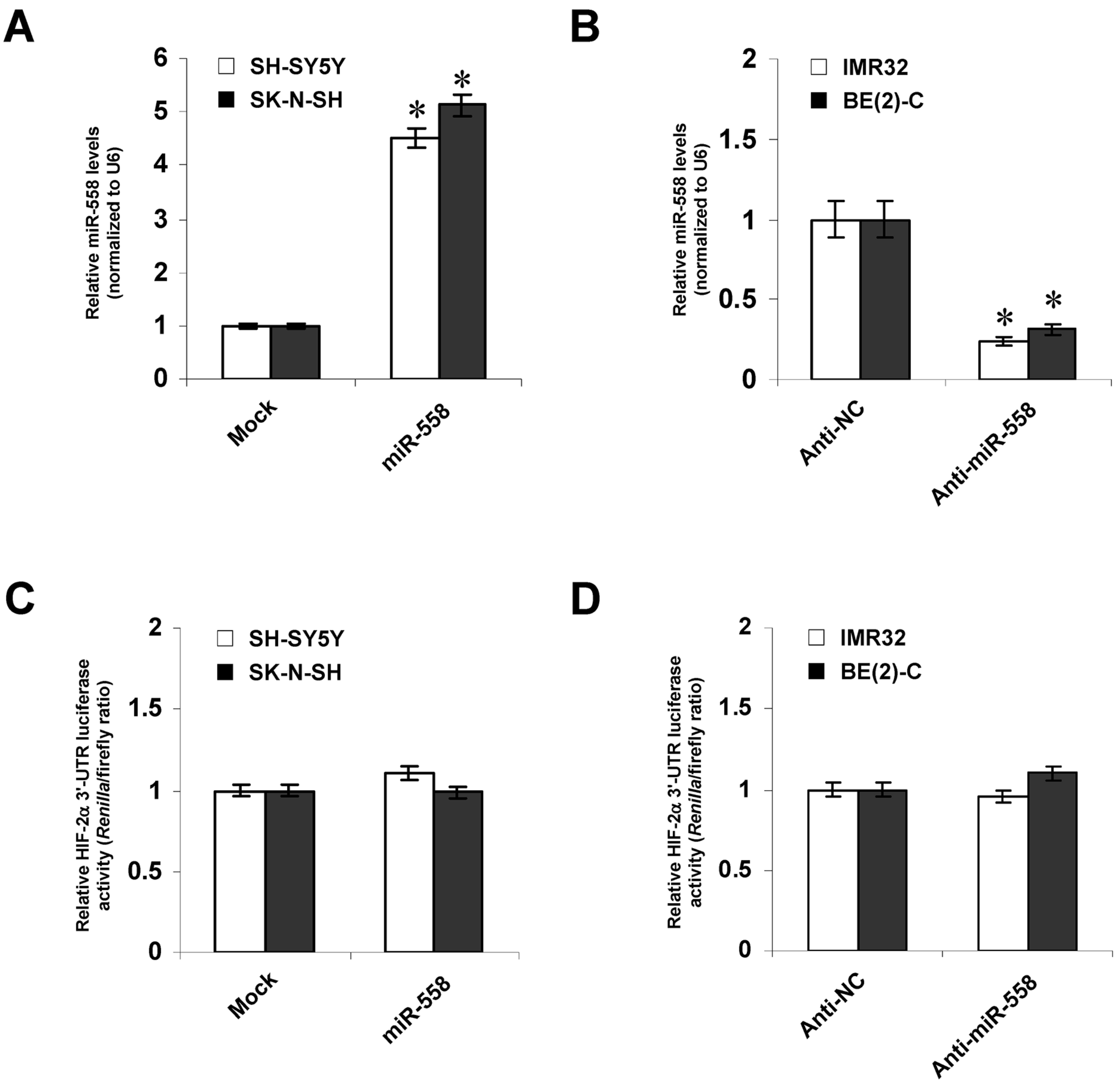


**Supplementary Figure S1: Effects of miR-558 on the 3'-UTR activity of *HIF-2α* in NB cells. A.** and **B.** real-time qRT-PCR showing the miR-558 levels in NB cells transfected with empty vector (mock), miR-558 precursor, anti-NC (100 nmol/L) or anti-miR-558 (100 nmol/L) inhibitors. **C.** and **D.** dual-luciferase assay indicating the activity of *HIF-2α* 3'-UTR luciferase reporter in NB cells transfected with mock, miR-558 precursor, anti-NC (100 nmol/L) or anti-miR-558 (100 nmol/L) inhibitors. * $P<0.01$ vs. mock or anti-NC.

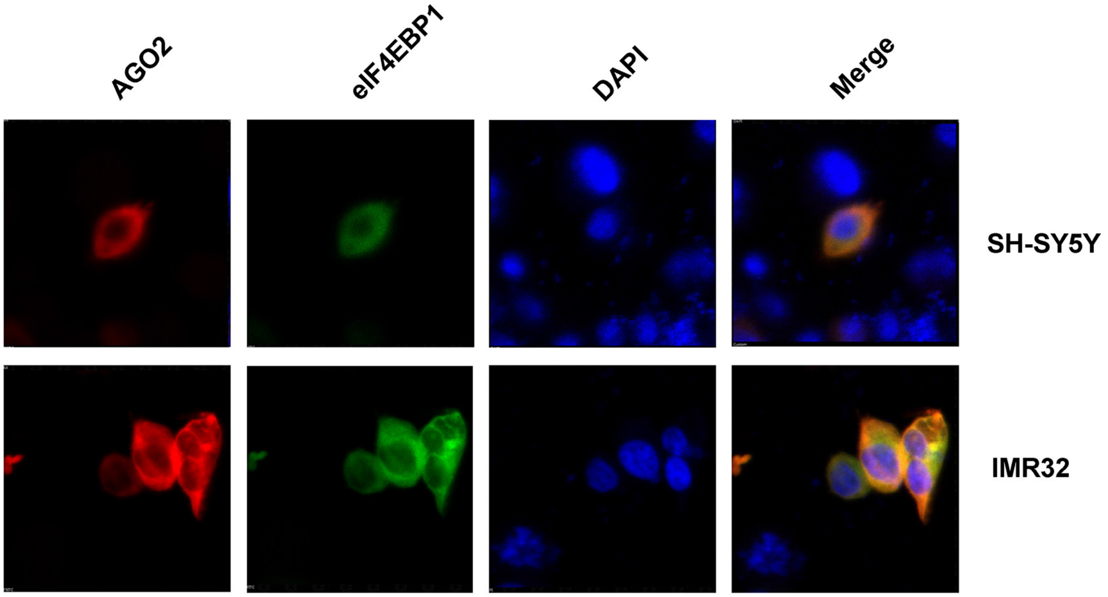


**Supplementary Figure S2: Endogenous expression of AGO2 and eIF4EBP1 in NB cells.** Immunofluorescence assay revealing the co-localization of AGO2 and eIF4EBP1 in cultured SH-SY5Y and IMR32 cells.

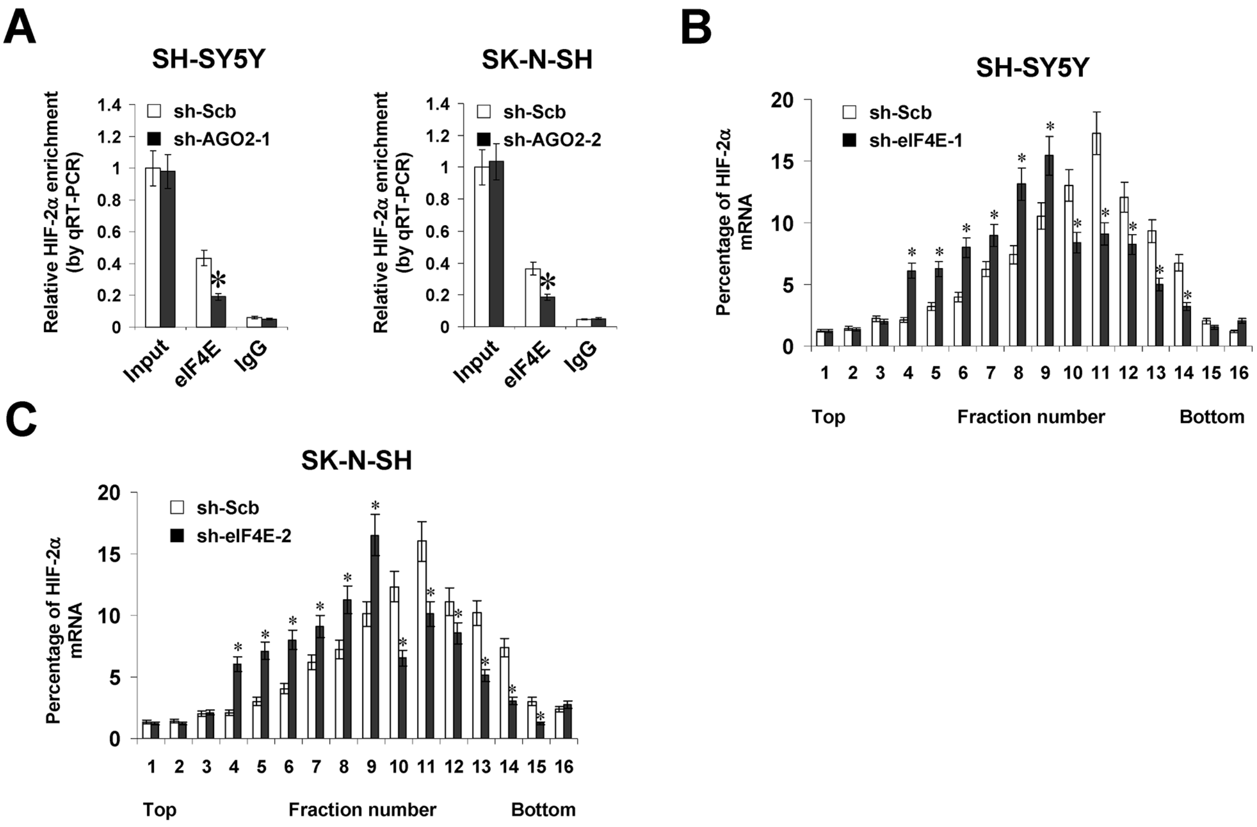


**Supplementary Figure S3: Effects of eIF4E on HIF-2α translation in NB cells. A.** RIP and real-time qRT-PCR assay showing the binding of eIF4E to *HIF-2α* 5'-UTR in NB cells transfected with sh-Scb or sh-AGO2. **B.** and **C.** sucrose gradient sedimentation assay indicating the distribution of *HIF-2α* transcripts to the polysome fractions in NB cells transfected with sh-Scb or sh-eIF4E. * $P<0.01$ vs. sh-Scb.

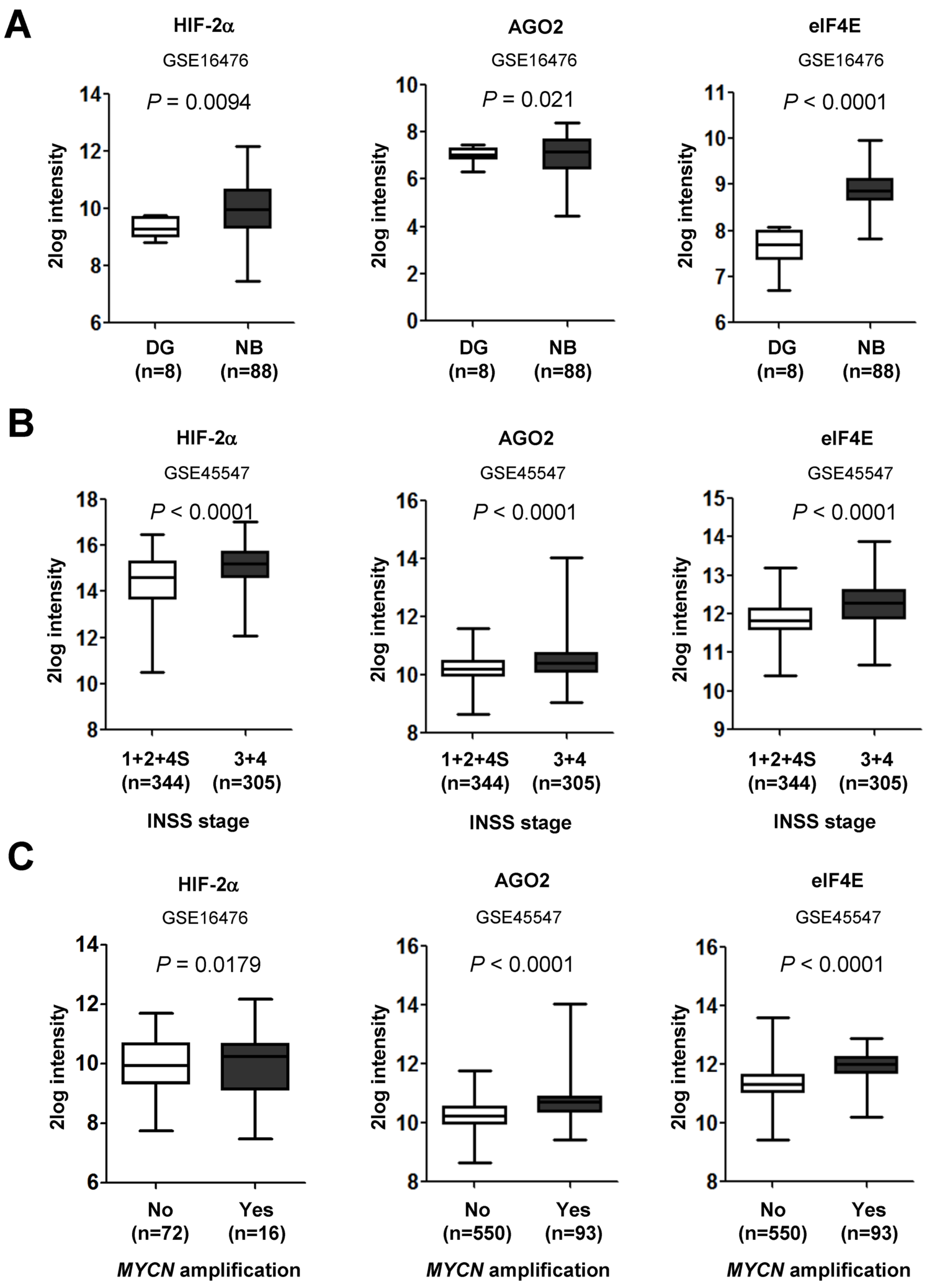


**Supplementary Figure S4: Expression of HIF-2α, AGO2, and eIF4E in public datasets. A.** the expression of HIF-2α, AGO2, and eIF4E in normal dorsal ganglia (DG) and NB tissues derived from GEO database and R2 microarray analysis and visualization platform (http://r2.amc.nl). **B.** and **C.** mining the GEO database and R2 microarray analysis and visualization platform (http://r2.amc.nl) showing the expression of HIF-2α, AGO2, and eIF4E in NB tissues with different INSS stages or *MYCN* amplification status.

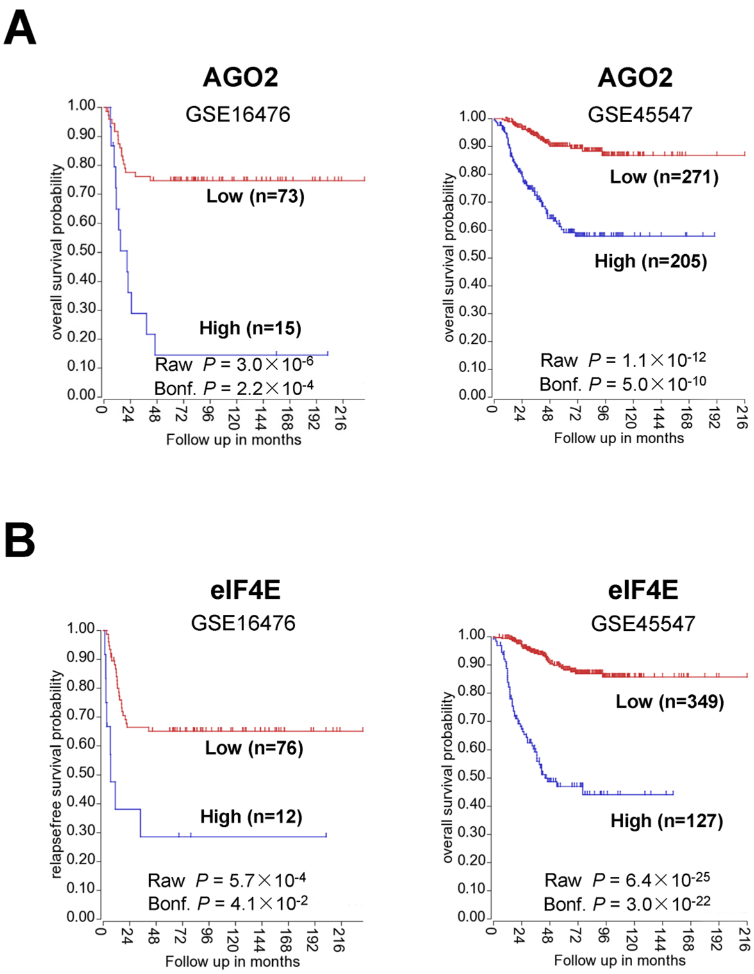


**Supplementary Figure S5: Patients' survival analysis.** Kaplan–Meier survival plots of NB cohorts (stratified by the scan method and adjusted by Bonferroni correction) derived from GEO database and R2 microarray analysis and visualization platform (http://r2.amc.nl) showing the survival probability of patients with high or low expression of AGO2 **A.** or eIF4E **B.**

**Supplementary Table S1: Primer sets used for qRT-PCR and RIP**

| Primer set | Primers | Sequence | Product size (bp) | Application |
|---|---|---|---|---|
| HIF-2α | Forward | 5’-AAAGCCTTGGAGGGTTTCATT-3’ | 292 | qRT-PCR |
| | Reverse | 5’-AGGTGGCTGACTTGAGGTTGA-3’ | | |
| HIF-2α 5’-UTR | Forward | 5’-AGGCGGCCGTACAATCCT-3’ | 187 | qRT-PCR, RIP |
| | Reverse | 5’-TGTCAGACCCGAAAAGAG-3’ | | |
| β-actin | Forward | 5’-ATCTACGAGGGGTATGCC-3’ | 227 | qRT-PCR |
| | Reverse | 5’-TAGCTCTTCTCCAGGGAG-3’ | | |
| miR-558 | Forward | RiboBio | | qRT-PCR |
| | Reverse | RiboBio | | |
| U6 | Forward | RiboBio | | qRT-PCR |
| | Reverse | RiboBio | | |

HIF-2α, hypoxia-inducible factor 2 alpha; 5’-UTR, 5’-untranslated region.

**Supplementary Table S2: Oligonucleotide sets used for constructs, inhibitors and short hairpin RNAs**

| Oligo set | Sequences |
|---|---|
| pre-miR-558 | 5'-TGCTGTGAGCTGCTGTACCAAAATGTTTTGGCCACTGACTGACATTTTGGTACAGCAGCTCA-3' (sense); |
| | 5'-CCTGTGAGCTGCTGTACCAAAATGTCAGTCAGTGGCCAAAACATTTTGGTACAGCAGCTCAC-3' (antisense) |
| pre-miR-NC | 5'-TGCTGAAATGTACTGCGCGTGGAGACGTTTTGGCCACTGACTGACGTCTCCACGCAGTACATTT-3' (sense); |
| | 5'-CCTGAAATGTACTGCGTGGAGACGTCAGTCAGTGGCCAAAACGTCTCCACGCGCAGTACATTTC-3' (antisense) |
| HIF-2α 5'-UTR mut (miR-558) | 5'-AGACTGTATGTTAGTACAGGCCCGGCCTCCGACTCCTTCCGACTCCCAGC-3' (sense); |
| | 5'-GGAGGCCGGGCCTGTACTAACATACAGTCTCAGGACACTGCCGAGGATTG-3' (antisense) |
| Anti-NC | RiboBio |
| Anti-miR-558 | RiboBio |
| sh-Scb | 5'-CCGGGCGAACGATCGAGTAAACGGACTCGAGTCCGTTTACTCGATCGTTCGCTTTTT-3'(sense); |
| | 5'-AATTCAAAAAGCGAACGATCGAGTAAACGGACTCGAGTCCGTTTACTCGATCGTTCGC-3' (antisense) |
| sh-AGO2-1 | 5'-CCGGGCACAGCCAGTAATCGAGTTTCTCGAGAAACTCGATTACTGGCTGTGCTTTTTG-3' (sense); |
| | 5'-AATTCAAAAAGCACAGCCAGTAATCGAGTTTCTCGAGAAACTCGATTACTGGCTGTGC-3' (antisense) |
| sh-AGO2-2 | 5'-CCGGCGTCCGTGAATTTGGAATCATCTCGAGATGATTCCAAATTCACGGACGTTTTTG-3' (sense); |
| | 5'-AATTCAAAAACGTCCGTGAATTTGGAATCATCTCGAGATGATTCCAAATTCACGGACG-3' (antisense) |
| sh-eIF4E-1 | 5'-CCGGCAGAGAGGAGGAAGGAGAAGTCTCGAGACTTCTCCTTCCTCCTCTCTGTTTTTG-3' (sense); |
| | 5'-AATTCAAAAACAGAGAGGAGGAAGGAGAAGTCTCGAGACTTCTCCTTCCTCCTCTCTG-3' (antisense) |
| sh-eIF4E-2 | 5'-CCGGCAGACCAGCAGATGGACAACTCTCGAGAGTTGTCCATCTGCTGGTCTGTTTTTG-3' (sense); |
| | 5'-AATTCAAAAACAGACCAGCAGATGGACAACTCTCGAGAGTTGTCCATCTGCTGGTCTG-3' (antisense) |
| sh-HIF-2α-1 | 5'-CCGGGCGCAAATGTACCCAATGATACTCGAGTATCATTGGGTACATTTGCGCTTTTTG-3' (sense); |
| | 5'-AATTCAAAAAGCGCAAATGTACCCAATGATACTCGAGTATCATTGGGTACATTTGCGC-3' (antisense) |
| sh-HIF-2α-2 | 5'-CCGGCAGTACCCAGACGGATTTCAACTCGAGTTGAAATCCGTCTGGGTACTGTTTTTG-3' (sense); |
| | 5'-AATTCAAAAACAGTACCCAGACGGATTTCAACTCGAGTTGAAATCCGTCTGGGTACTG-3' (antisense) |

pre-miR-NC, negative control miRNA precursor; HIF-2α, hypoxia-inducible factor 2 alpha; 5'-UTR, 5'-untranslated region; Anti-NC, negative control inhibitor; sh-Scb, scramble short hairpin RNA; AGO2, Argonaute 2; eIF4E, eukaryotic translation initiation factor 4E.
